# Deep brain imaging of three participants across 1 year: The Bergen breakfast scanning club project

**DOI:** 10.3389/fnhum.2022.1021503

**Published:** 2022-10-17

**Authors:** Meng-Yun Wang, Max Korbmacher, Rune Eikeland, Karsten Specht

**Affiliations:** ^1^Department of Biological and Medical Psychology, University of Bergen, Bergen, Norway; ^2^Mohn Medical Imaging and Visualization Centre, Haukeland University Hospital, Bergen, Norway; ^3^Department of Health and Functioning, Western Norway University of Applied Sciences, Bergen, Norway; ^4^Department of Education, UiT The Arctic University of Norway, Tromsø, Norway

**Keywords:** deep neuroimaging, precision brain mapping, resting-state fMRI, MRS, reliability, variability, reproducibility

## Abstract

Our understanding of the cognitive functions of the human brain has tremendously benefited from the population functional Magnetic Resonance Imaging (fMRI) studies in the last three decades. The reliability and replicability of the fMRI results, however, have been recently questioned, which has been named the replication crisis. Sufficient statistical power is fundamental to alleviate the crisis, by either “going big,” leveraging big datasets, or by “going small,” densely scanning several participants. Here we reported a “going small” project implemented in our department, the Bergen breakfast scanning club (BBSC) project, in which three participants were intensively scanned across a year. It is expected this kind of new data collection method can provide novel insights into the variability of brain networks, facilitate research designs and inference, and ultimately lead to the improvement of the reliability of the fMRI results.

## Introduction

The pursuit of understanding how the brain works has been driving researchers to develop innovative and novel utilities. Since the discovery of functional Magnetic Resonance Imaging (fMRI) in the early 90s (Kwong et al., 1992; Ogawa et al., 1992) it has been ubiquitously utilized in the cognitive neuroscience research field due to its relatively high spatial resolution compared to other non-invasive neuroimaging tools. Undoubtedly, by leveraging fMRI, our understanding of the brain has been substantially deepened and expanded. For example, it is now well known that there are several brain regions preferentially responding to specific stimuli, such as the fusiform area (FFA) for faces (Kanwisher et al., 1997), the visual word form area for words (Nobre et al., 1994), and the number form area (NFA) for numbers (Shum et al., 2013). Besides, several brain networks have been consistently recognized with the resting-state fMRI data (Damoiseaux et al., 2006), which can be corresponded to the brain networks at tasks (Smith et al., 2009). In addition, the association between the brain indices and phenotypes has also been widely explored (Genon et al., 2018; Masouleh et al., 2019).

Although new insights and perspectives have been proliferated with fMRI data, however, the reliability and reproducibility have been recently questioned (Bennett and Miller, 2010). It has shown that different parameter choices in the processing pipeline could affect the final conclusions (Botvinik-Nezer et al., 2020). What’s more, the puzzlingly high correlation in fMRI studies in emotion, personality, and social cognition (Vul et al., 2009) is somehow worrisome [but also see different opinion (Lieberman et al., 2009)]. Playing into this point, low statistical power, often produced by small sample sizes, can undermine the reliability of the neuroscience research (Button et al., 2013).

Having said that, how can we increase the reliability and reproducibility of fMRI studies, especially about the brain-behavior correlation? In one recently published article in *Nature*, the researchers have suggested that acquiring thousands of individuals’ data can be one way to reliably detect small effects from brain-wide associations (Marek et al., 2022). A corresponding opinion article (Gratton et al., 2022) has suggested that there are generally two ways to increase reliability by obtaining sufficient data: “going big” as in the mentioned article, or “going small” in which several individuals can be repeatedly scanned to form a deep neuroimaging dataset.

The concept of deep brain imaging is relatively new but not exotic. It can be traced down to the MyConnectome project (Laumann et al., 2015; Poldrack et al., 2015; Poldrack, 2021), where a single subject was densely scanned for around 2 years resulting in around 100 MRI sessions. It has been shown that the brain networks trend differently between group level and individual level, and different factors such as drinking coffee can introduce connectomic shifts (Poldrack et al., 2015). The Bergen breakfast scanning club (BBSC) project is inspired by the MyConnectome project, and we would like to replicate the findings from the MyConnectome project as well as explore other factors which could affect fMRI signals, for example, the effect of seasonal long-night and long-day phenomena which are unique to world regions close to the poles.

Based on this endeavor, here we report a “going small” project implemented in our department between February 2021 and 2022, where three individuals were densely scanned for a year. All three subjects are male, right-handed, and speak at least two languages (age: 31, 27, and 40). The name of the project is a combination of scanning place Bergen and the breakfast club, where initially we wanted to scan the participants in the morning. It was also an homage to the Midnight Scanning Club dataset (Gordon et al., 2017).

## Project’s description

The overarching goal of the BBSC project is to understand the variability of the fMRI signal and explore precision brain mapping at the individual level. The purpose of the project is threefold. The first is to explore the effects of exogenous factors exerting on fMRI signals, such as time of day, and time of year since the long-night and long-day phenomena in Norway. The second is to explore how endogenous factors affect fMRI signals, such as emotional state, physiological indices, and daily routine. The third is to explore the relationship between functional and structural brain organization. Accordingly, there were three protocols in the project including a behavioral, a functional, and a structural protocol. Brain imaging data were acquired at the Haukeland University Hospital with an up-to-date high-performance 3T MRI (GE Discovery 750).

It was expected that the participants would be scanned twice a week (once in the morning, once in the afternoon) with the functional protocol and once a month with the structural protocol, which would result in 50 scanning sessions in total for each participant.

In the behavioral protocol, we have collected information about the participants’ daily life such as sleep duration, time spent on excise, and any significant daily events. The behavioral protocol was implemented twice a day (after getting up and before going to bed) on participants’ mobile phones.

**FIGURE 1:**
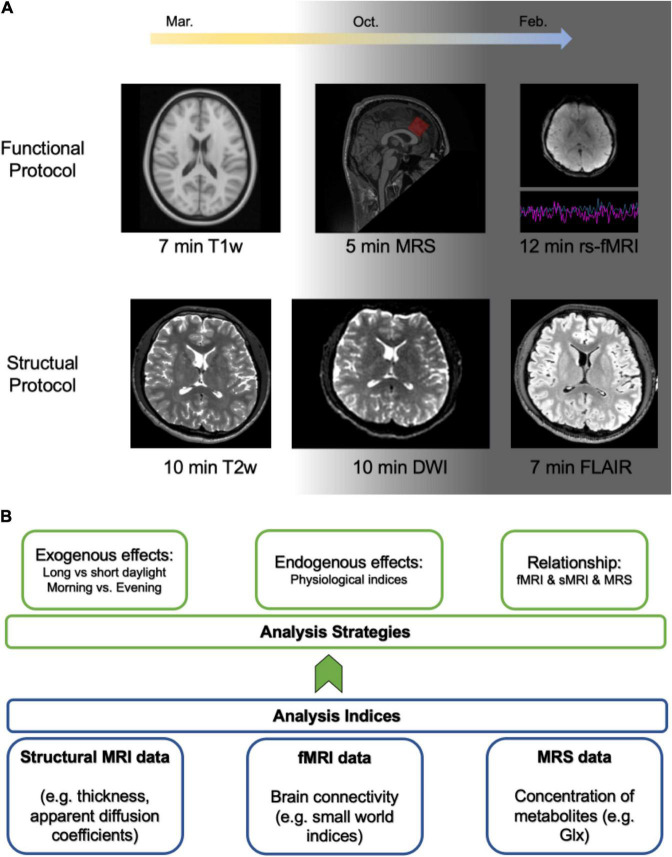
Skeleton of the project and analysis plan. **(A)** The project neuroimaging protocols. The background indicates that daylight time will gradually be minimized from spring to winter. The functional protocol was implemented twice a week while the structural protocol was implemented once a month. **(B)** The illustration of the analysis plan.

In the functional protocol (Figure 1A), first, 7 min T1-weighted brain image data were collected, which will be used for brain registration and gray matter changes exploration. Second, to test the metabolism level, 5 min magnetic resonance spectroscopy (MRS) data were recorded. MRS can detect metabolism changes in the brain, where major metabolites concentration can be calculated (Harris et al., 2017). Last, 12 min resting-state fMRI data as recommended (Birn et al., 2013) have been collected as well as the pulse, respiratory, and blood oxygen saturation levels. The resting-state fMRI, simply put, records systematic, non-random variations in the activation of the brain in the absence of a specific task. These activations are indirectly reflected in regional fluctuations in the level of oxygenation of the blood (a.k.a. BOLD signal). It has been widely used to explore functional brain activation (Biswal et al., 1995), from which brain networks (also called “resting-state networks”) can be extracted (Damoiseaux et al., 2006) and network-related characteristics can be explored (Bullmore and Sporns, 2009; Van Den Heuvel and Pol, 2010). Questionnaires including the outside and inside weather indices and the participants’ emotional states were filled out before scanning, while the Amsterdam resting state questionnaire (Diaz et al., 2013) was filled out after scanning. This protocol was implemented twice a week for a year except for public holidays.

In the structural protocol (Figure 1A), 10 min T2-weighted brain images were first collected to examine the variability of the gray matter. Then, 10 min diffusion-weighted imaging (DWI) and 7 min Fluid-attenuated inversion recovery (FLAIR) brain imaging data were recorded to explore the variability of the white matter. This protocol was implemented once a month for a year except for public holidays.

## Analysis plan

The overview of the analysis plan was depicted in Figure 1B. First and foremost, we will chart the fluctuation of the fMRI data and MRS data to depict the variability of those signals over a year. Second, in order to achieve the first purpose, indices extracted from the neuroimaging data will be compared between season-dependent long and short daylight periods, as well as morning and evening. Third, in order to get the second purpose, we will explore the relationship between physiological data (pulse, respiratory, and blood oxygen saturation levels) and resting-state fMRI data. Accordingly, we will propose how to clean the resting-state fMRI data with these physiological data. Finally, in order to reach the third purpose, a virtual brain model will be constructed based on the virtual brain (TVB) platform (Sanz Leon et al., 2013). The dataset will be made public after the main results have been published.

## Discussion

In a conventional way, dozens or hundreds of participants should be recruited for an fMRI study or big open datasets can be leveraged to explore the questions that one is interested in. Contrary to the conventional way, here we report a deep brain imaging project, the BBSC project, where three participants have been densely scanned for a year. The relatively new data collection method can provide new insights into the architecture of the brain and the variability of how the brain signal fluctuates over a year.

Deep brain imaging data can extend our understanding of brain parcellation which are yet mainly based on the “going big” approach. Brain templates or parcellations are conventionally constructed from congregated data from different participants, which inevitably will wipe out the individual characters. For example, a recent study shows that a brain region belonging to the default mode network (DMN) network at the group level brain parcellation is actually affiliated with the attention network at the individual level (Laumann et al., 2015). By leveraging deep brain imaging data, detailed brain parcellation based on individual functional connections can be revealed (Gordon et al., 2017; Marek and Greene, 2021), which can further make a contribution to precision medicines (Gratton et al., 2020). The BBSC project will advance this endeavor, and further based on the individual brain parcellation explore the variability of individual brain networks.

Furthermore, deep brain imaging data can better illustrate the relationship between structural and functional brain organizations. By using the functional and structural data collected in the BBSC project, a virtual brain model can be constructed with the TVB platform (Sanz Leon et al., 2013). Thus, the dependence between functional and structural brain organizations can be illustrated by manipulating the parameters in the model (Sanz Leon et al., 2013). Overall, the deep brain imaging method holds much potential and can advance our understanding of how the brain works (Gratton and Braga, 2021).

Deriving from the project, we can provide practical information for fMRI data collection and analysis. For example, it could be ideal that participants should be scanned within a season or daytime window; always measure variable X in addition to fMRI because the signal can be convoluted by variable X. In summary, the rich data collected from the BBSC project entails the potential to chart the variability of the fMRI signal over a year, better comprehension of the relationship between functional and structural data, and the dependence between exogenous and endogenous factors and fMRI signals. Hereafter, the results can enhance the reliability of the fMRI studies.

## Data availability statement

The original contributions presented in this study are included in the article/supplementary material, further inquiries can be directed to the corresponding authors.

## Ethics statement

The studies involving human participants were reviewed and approved by the Regional Committee for Medical and Health Research Ethics. The patients/participants provided their written informed consent to participate in this study.

## Author contributions

M-YW, MK, RE, and KS contributed to the conception of the research project and final version of the manuscript. All authors contributed to the article and approved the submitted version.
